# On the Study of Deubiquitinases: Using the Right Tools for the Job

**DOI:** 10.3390/biom12050703

**Published:** 2022-05-14

**Authors:** Cody Caba, Azam Mohammadzadeh, Yufeng Tong

**Affiliations:** Department of Chemistry and Biochemistry, University of Windsor, Windsor, ON N9B 3P4, Canada; cabac@uwindsor.ca (C.C.); mohamm57@uwindsor.ca (A.M.)

**Keywords:** deubiquitinase (DUB), ubiquitin (Ub), ubiquitin variant (UbV), activity-based probes (ABP), inhibitors

## Abstract

Deubiquitinases (DUBs) have been the subject of intense scrutiny in recent years. Many of their diverse enzymatic mechanisms are well characterized in vitro; however, our understanding of these enzymes at the cellular level lags due to the lack of quality tool reagents. DUBs play a role in seemingly every biological process and are central to many human pathologies, thus rendering them very desirable and challenging therapeutic targets. This review aims to provide researchers entering the field of ubiquitination with knowledge of the pharmacological modulators and tool molecules available to study DUBs. A focus is placed on small molecule inhibitors, ubiquitin variants (UbVs), and activity-based probes (ABPs). Leveraging these tools to uncover DUB biology at the cellular level is of particular importance and may lead to significant breakthroughs. Despite significant drug discovery efforts, only approximately 15 chemical probe-quality small molecule inhibitors have been reported, hitting just 6 of about 100 DUB targets. UbV technology is a promising approach to rapidly expand the library of known DUB inhibitors and may be used as a combinatorial platform for structure-guided drug design.

## 1. Introduction

Over 40 years ago, the seminal works of Avram Hershko, Aaron Ciechanover, and Irwin Rose (and, later, Alexander Varshavsky) engendered the ubiquitin (Ub) field as we know it today. A first-hand account of these early years, for which the 2004 Nobel Prize in Chemistry was awarded, has been delineated by Varshavsky [1]. It is now well-understood that ubiquitination is a tightly regulated and highly specific post-translational modification (PTM) involved in seemingly all biological processes. Covalent attachment of the small 76-amino acid Ub to a protein’s lysine ε-amine or N-terminal α-amine, via an iso- or α-peptide bond, respectively, is diverse in consequences. Proteasome-targeted degradation is the canonical role of this modification, but countless studies have shed light on its criticality for signal transduction, trafficking, gene expression, and other cellular processes [2].

The complexity of molecular outcomes as a result of ubiquitination presents a ubiquitin code [3,4]. Since Ub itself can be ubiquitinated at any of its seven lysine residues (K6, K11, K27, K29, K33, K48, and K63), as well as its N-terminus (M1), in theory, this modification is architecturally limitless. Adding another layer of complexity is the fact that Ub can also be post-translationally modified by phosphorylation, as in the case of phospho-Ser-65 during mitophagy [5,6,7,8,9] or the phospho-Thr-12 for DNA damage response [10], lysine acetylation for inhibiting Ub chain elongation [11], and by Ub-like modifiers (Ubls), for which the physiological significance is not well-known [2,12]. The encoding and decoding of the ubiquitin code have been an intense subject of research. Our cumulative knowledge has allowed us to exploit the writers, readers, and erasers of the Ub machinery for novel therapeutics in the fight against cancer [13], aging-associated diseases [14], intellectual disorders [15], and more.

### 1.1. Deubiquitinase Enzymes: Classification and Activity Regulation

**Figure 1:**
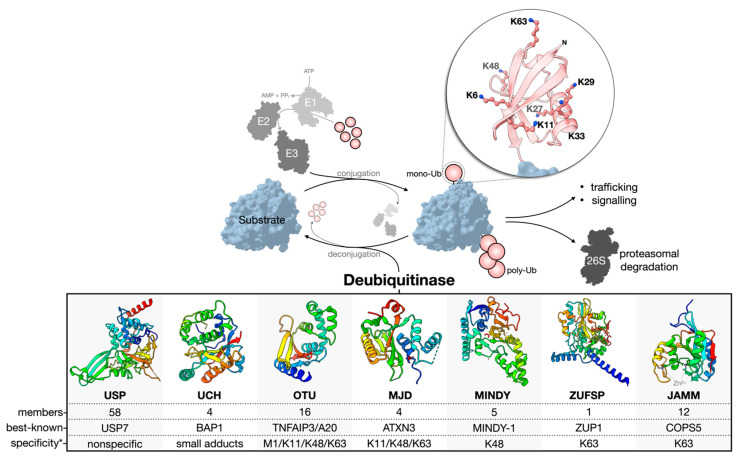
Ub conjugation and deconjugation. Ubiquitination is an ATP-dependent process orchestrated by an E1–E2–E3 enzymatic cascade utilizing free, mono-Ub. The N-terminus or any of the seven lysine residues of Ub can be ubiquitinated to facilitate poly-Ub chain assembly following the sequential rounds of conjugation. The removal or deconjugation of Ub is then mediated by DUBs, for which seven families have been classified. The structure of the DUB catalytic domain of a representative family member is shown: USP, USP7 pdb 4m5w [48]; UCH, UCH-L3 pdb 1uch [49]; OTU, OTUD1 pdb 4bop [25]; MJD, Jos-2 pdb 6pgv [50]; MINDY, MINDY-1 pdb 5jqs [41]; ZUFSP, ZUP1 pdb 6ei1 [51]; JAMM, STAMBPL1 pdb 7l97 [52]. The most well-known member of each family is presented based on the number of publications curated from the NCBI Entrez database. * The reader should be aware that reported Ub chain specificities are assay-dependent, and many exceptions exist.

Ubiquitination is reversed by the activity of deubiquitinase enzymes (DUBs) (Figure 1). About 100 human DUBs are known, and they are divided into seven major families: the cysteine proteases of the USP (ubiquitin-specific proteases), UCH (ubiquitin C-terminal hydrolases), OTU (ovarian tumor), MJD (Machado-Joseph domain-containing proteases), MINDY (motif interacting with the Ub-containing novel DUB family), and ZUFSP (zinc finger with the UFM1-specific peptidase domain protein) families and the Zn-dependent metalloproteases of the JAMM (JAB1/MPN/MOV34 domain-associated) family [4,16,17,18,19]. The USP family is the largest, with 58 constituents. The members of each family are related through structurally homologous catalytic domains but can vary greatly by the presence of insertions, deletions, and additional domains serving diverse functions. Cysteine protease DUBs utilize a catalytic triad or dyad to facilitate a nucleophilic attack and hydrolysis of the scissile bond connecting the C-terminal Gly-76 of Ub to a substrate or other Ub of a poly-Ub chain. Contrarily, zinc-dependent metalloprotease DUBs (metalloDUBs) utilize a coordinated zinc ion and an activated, nucleophilic water molecule for catalysis [4].

DUBs are essential for maintaining Ub homeostasis by processing Ub precursors (UBC, UBB, UBA52, and UBA80) [20] and unconjugated chains [17]; recycling Ub at the proteasome [21]; and deubiquitinating various targets. Additionally, they function as interpreters and editors of a highly complex code. E3-DUB complexes are common and couple conjugation and deconjugation to facilitate chain editing and fine-tuning of the molecular output [22]. In the rare case of A20 (an OTU family member), both E3 and DUB activities are encoded into a single multifunctional enzyme [23].

The factors dictating DUB activity and substrate specificity include the structure of the catalytic domain, the presence of additional domains for substrate recruitment or Ub binding [24,25,26], alternative splicing [27], allostery [28], oligomerization [29], PTMs [30], and subcellular localization [4]. Importantly, not all DUBs are catalytically active. Approximately 10% are pseudo-enzymes currently found in the USP, OTU, JAMM, and MINDY families [31]. Pseudo-DUBs are critical components of large macromolecular complexes or as allosteric modulators of active DUBs. Most famously, PSMD7, a pseudo-DUB, dimerizes with the active DUB PSMD14. This complex is a critical component of the 19S proteasome regulatory particle [32,33,34]. Without detectable activity, pseudo-DUBs are most readily identified by sequence and structural similarity to catalytically competent homologs.

DUBs can discriminate the linkage type, chain length, modifier (Ub or Ubl), and/or the substrate to which Ub is conjugated [35]. Furthermore, they can remove poly-Ub chains in different modes: iteratively at the distal end (*exo*-cleavage), from within a chain (*endo*-cleavage), or all at once (*en bloc*-cleavage) [36]. Members of the USP family are generally linkage-nonspecific and often referred to as promiscuous [24,37,38]; however, using tool reagents, including small molecule compounds and activity-based probes, our group and others have revealed surprising linkage-dependent processing activities for USP9X [39] and USP7 [40], two USPs previously believed to be nonspecific. Whether or not other members of the USP family have a similar specificity remains to be explored. Contrarily, members of the OTU, MINDY, ZUFSP, and JAMM families tend to be linkage-specific [4,17,25,38,41,42,43,44,45]. For OTUD1, a C-terminal Ub-interacting motif (UIM) endows specificity for K63 linkages by forming a proximal Ub-binding site (S1’) to orient the scissile bond across the catalytic site [25]. The structural determinants of the linkage specificity can also be leveraged for chain length specificity. The five Ub-binding sites of the catalytic domain of MINDY1/2 render strict K48 linkage specificity and the ability to sense the poly-Ub chain length to modulate the *exo*- (<6 Ub) and *endo*-cleavage (>5 Ub) modes [46]. UCH DUBs preferentially cleave Ub from small or unstructured C-terminal leaving groups. This represents a sort-of substrate specificity that results from an active site crossover loop limiting the size of ubiquitinated substrates able to occupy the catalytic cleft [47].

### 1.2. Challenges and Missteps When Studying DUBs at the Cellular Level

Since ubiquitination is involved in almost all biological processes, including protein homeostasis, transcription regulation, DNA repair, endocytosis and endolysosomal sorting, autophagy, immune response, and stem cell renewal, unsurprisingly, DUBs are also major players in these diverse cellular functions. Therefore, their dysregulation is associated with many diseases. This has been the subject of multiple recent reviews, either about their general functions [17,18,53] or on specific disease areas such as neuronal [54], cardiovascular [55], developmental [56], autoimmune [57] diseases, and cancers [58,59] and is beyond the scope of this review. Instead, we highlight the importance of using quality tool reagents when exploring the biology of DUBs at the cellular level.

Due to the scarcity of quality DUB inhibitors, studies often use unvalidated, weak, or semi-selective inhibitors when probing for cellular functions. These practices can lead to spurious conclusions and add to the reproducibility crisis [60,61]. For example, compounds WP1130 and G9 have been used to examine the biological roles of USP9X, a large, 250-kDa USP family DUB. Studies using these compounds have claimed to elucidate numerous interactors and functions [62]; however, many of these interactors (for example, E3 ligases ITCH [63] and FBXW7 [64]; Halabelian and Tong unpublished data) and functions (Michael Clague personal communication) cannot be validated. One inhibitor, G9, which cross-reacts with USP5 and USP24 [65], has been used by many separate studies to assess the functions of USP9X [66,67,68], USP5 [69,70], or USP24 [71]. It is difficult to exclude the possibility of off-target effects, crosstalk, or functional compensation between these DUBs. On the other hand, FT709, a probe-quality inhibitor with a nanomolar affinity for USP9X, has provided much clearer evidence of its involvement in ribosomal stalling and confirmed its interaction with two other E3 ligases, ZNF598 and MKRN2 [72]. Another example is spautin-1 [73], which is widely used as a specific autophagy inhibitor for its inhibition of both USP10 and USP13. These two DUBs have very different structures. Whereas USP10 has a typical USP fold without insertions, USP13 has two ubiquitin-associated (UBA) domains inserted in the catalytic domain. USP13 is closer in sequence and domain architecture to USP5 than USP10. No orthogonal biophysical methods, such as the surface plasmon response (SPR) or isothermal titration calorimetry (ITC), were used to validate the binding of spautin-1 with USP10 or USP13. Furthermore, our unpublished data could not recapitulate the inhibition of USP10 by spautin-1. Instead, a ubiquitin variant (UbV) was later shown to be an inhibitor of USP10 both in vitro and in vivo [74]. Recently, we also confirmed that a small molecule inhibitor for STAMBP, BC–1471, which has been used to study the role of this JAMM family DUB in regulating the inflammasome constituent NALP7 [75], does not inhibit STAMBP in vitro [52]. The lesson is that many studies are overly enthusiastic about testing the potency of DUB-modulating compounds in vivo without first rigorously validating the target engagement and off-target effect in vitro. Caution must be taken when selecting an inhibitor, modulator, or tool molecule for studying DUBs in a complex cellular context.

The expansion of available pharmacological modulators and chemical probes [76,77,78] helps to continually further our understanding of DUBs. This review aims to provide researchers entering the field of ubiquitination with knowledge of the available pharmacological modulators and tool molecules. We begin by examining the toolbox of probe-quality small molecule inhibitors, engineered ubiquitin variants (UbVs), and activity-based probes (ABPs). Their design and use for interrogating DUB activity in vitro are discussed briefly. Moreover, we shed light on how these tools are leveraged for understanding DUB biology at the cellular level in conjunction with other technologies, such as proteomics and genetic screens.

## 2. Small Molecule DUB Inhibitors

Despite a decade of intensive investment from industry and academic labs, most reported DUB inhibitors have a low binding affinity in the micromolar range and lack selectivity. This was demonstrated by a comprehensive profiling study using the MALDI-TOF mass spectrometry of 11 inhibitors against 42 DUBs [37] and by Medivir’s DUB platform [79]. ML323, an inhibitor of the USP1/UAF1 complex, was developed by Zhuang Lab [80,81] and stood to be the only USP family DUB inhibitor that met the chemical probe criteria [82] by 2016. However, the exact structural details on how ML323 binds to the USP1/UAF1 complex remained elusive, which prevented further structure-based optimization of the inhibitor. When several groups developed a series of high-quality, structurally defined USP7 inhibitors, the efforts in DUB inhibitor discovery were reinvigorated. The progress on USP7 inhibitors was nicely summarized in Pozhidaeva and Bezsonova’s review [83]. The demonstration of the druggability of USPs and the identification of structurally defined DUB inhibitors opened many opportunities to pharmacologically interfere with their functions in vivo and to explore their biology in disease states. The advances have been the subject of several recent excellent reviews [77,84,85,86] and will not be repeated here. We would, however, like to point out that some low-quality compounds, such as the now-invalidated BC-1471 [75], are still included in one of the most comprehensive reviews [86].

In the last several years, the chemical biology community has been advocating a stringent evaluation of chemical tool reagents for the study of the biology of protein targets [76,77,78]. These criteria include <100 nM in vitro potency, >30-fold selectivity against other members of the same protein family, off-target profiling, and cellular on-target effects at <1 ¦ÌM. A combination of orthogonal validation methods to confirm target engagement is essential, especially considering the various shortcomings of some popular inhibitors (discussed above).

We have kept track of the high-quality chemical inhibitors of DUBs using a publicly accessible repository called UbiHub [87]. So far, only four USPs (USP7, USP1, USP9X, and USP30); one UCH (UCHL1); and one JAMM family DUB (CSN5) have chemical probe-quality small molecule inhibitors (Table 1). However, it is noteworthy that some of these are covalent inhibitors, such as FT385 for USP30 [88] and IMP–1710 for UCHL1 [89,90], which both utilize a cyanopyrrolidine warhead for modifying the catalytic cysteine residue. Despite their selectivity over other DUBs, they may have off-target reactivity towards other unrelated enzymes, such as dehydrogenases, as demonstrated for the UCHL1 inhibitor MT16-205 [89]. Thus, extra care must be exercised when using these inhibitors to probe the biology of the intended DUB targets. The best practice for small molecule inhibitors in studying the biology of protein targets is well-established [91]. Readers are encouraged to follow the do’s and don’ts when using chemical probes to obtain credible results.

## 3. Ubiquitin Variants: A Combinatorial Approach to Selective DUB Targeting

Developing small molecule DUB binders of chemical probe quality [82] is a major hurdle. USP7 inhibitors are a testament to this; a tour de force of more than a decade [95,100]. As a result, most DUBs have yet to be successfully targeted. Certain characteristics provide evidence of their poor tractability: (1) large, shallow interaction interfaces; (2) catalytic pockets either ill-defined or difficult to differentiate between homologs; and (3) highly plastic and cryptic structures governed by extensive allosteric mechanisms in response to various stimuli [28,36,101,102]. Fortunately, the molecular details that underpin the activity of a given DUB can be unique and therefore exploited by selective modulators [77].

**Figure 2:**
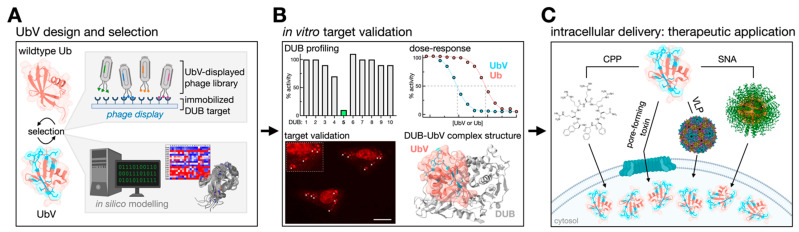
The pipeline of UbV development. (**A**) Wildtype Ub is randomly mutated to generate a library of UbVs that are subject to iterative rounds of screening to identify target binders, usually by phage display and/or computational methods. (**B**) The selectivity and affinity of promising leads are validated in vitro using orthogonal methods, such as profiling against a panel of homo- or heterologous targets, quantifying inhibitory concentrations by an enzyme assay, determining intracellular targets by immunofluorescence or MS, and solving the structure of UbV-bound targets. (**C**) UbVs of probe-quality potency can be examined in vivo for their therapeutic potential. A variety of cell delivery methods can facilitate the transport of purified UbVs to the intracellular space. CPP, cell-penetrating peptide; SNA, spherical nucleic acid; VLP, virus-like particle.

The paucity of reliable small molecule modulators for DUBs led the Sidhu group to develop a protein engineering and phage display platform to generate UbVs able to interrogate the landscape of the Ub proteasome system (UPS) (Figure 2A) [103]. Using Ub as a scaffold, massively randomized libraries of UbVs were screened against a panel of DUB targets for enhanced affinity and selectivity. The strategy is combinatorial and has the capacity to outperform the throughput of small molecule screens [104]. The surface of Ub centered on the Phe-4, Ile-36, and Ile-44 patches [3] is a conserved core functional epitope important for molecular recognition [105,106,107]. Soft randomization of the regions allows for tailoring interaction characteristics with Ub-binding proteins [103]. Therefore, UbVs stand to gain both affinity and selectivity. Affinity enhancements can be achieved, because Ub-binding proteins, including DUBs, have an intrinsically low affinity for Ub that is in the micromolar range [41,108,109]. Based on the strict conservation of the Ub sequence, low-affinity interactions are likely evolutionarily constrained—in some cases, preventing substrate trapping relative to the high intracellular Ub concentration. Selectivity enhancements are possible, because the Ub-binding surfaces of homologous DUB catalytic domains, despite being topologically similar, are divergent in the sequence. This means unique Ub-binding hotspot residues may be exploited by new Ub sequences. In their pioneering work, the Sidhu group produced a variety of competitively inhibiting UbVs with a nanomolar affinity and selectivity for USP2, USP8, USP21, and OTUB1. Less well-characterized UbVs were also reported for the JAMM family DUB BRISC, the E2 Cdc34, and the E3s NEDD4 and ITCH [103].

Since inception, UbVs have been developed for almost every level of the UPS. Although binders for the human E1 Ub-activating enzymes have not yet been reported, UbVs targeting Ub-conjugating components at the E2 [103,110,111] and E3 [103,112,113,114,115,116,117] levels are established. Some UbVs can even bind select UBDs [117,118,119], thus broadening the scope of this technology to include readers of the UPS not directly involved with conjugation or deconjugation. UbVs targeting human DUBs of the USP [74,103,117,120,121,122], UCH [123,124], OTU [103,117], JAMM [52,103], and MJD [117] families are known (Table 2), as well as for viral DUBs [125]. Ub has a β-grasp fold that is found in many protein–protein interaction (PPI) modules [126]. We postulate that the UbV technique can be applied to generate binders for many protein families, but it has a unique advantage towards DUBs, since Ub is the natural substrate and has a large interaction interface.

The site to which a UbV binds dictates its function as an inhibitor, activator, or allosteric modulator. This has been nicely demonstrated for the UbVs targeting the HECT family of E3 ligases [112]. To date, most DUB-targeting UbVs are competitive inhibitors that bind the catalytic site. A potential noncompetitive mechanism is observed for UbVs that bind USP15 and only partially occlude the Ub-binding site. The interaction stabilizes the inactive conformation akin to an apo-USP closed hand, thereby blocking the substrate accessibility [120]. Furthermore, some UbVs can bind to alternative sites, such as DUSPs (domains found in Ub-specific proteases) of multiple USP family members [120,127], the UBD of USP37 [103], the Ubl domains of USP15 [120], or the UIMs in the large insertion of the USP37 catalytic domain [121]. To interrogate the catalytic mechanism of USP37, a UbV binding UIMs 1–3 was shown to inhibit the cleavage of K48 di-Ub in vitro, while a UbV specific to only UIM1 had no effect. The authors suggest this points to a role for UIM2 and UIM3 in the interaction with the proximal Ub of a poly-Ub chain [121]. Veggiani et al. recently developed UbVs targeting 42 diverse UIMs. Among them, UbV.28 was able to bind USP28 and inhibit the hydrolysis of the poly-Ub chains but not Ub-AMC, a mono-Ub fluorogenic substrate lacking a proximal Ub [117]. Like UbV.37.1, these data show UbVs can be designed to inhibit specific DUB activities by binding alternative sites. Furthermore, together with the Zhang group, we developed and characterized UbVs specific to the JAMM family paralogs STAMBP and STAMBPL1. Our analyses provided first-in-class structural information about the determinants of UbV selectivity for this sole family of metalloDUBs [52]. UbV^SP.3^ was >250-fold more specific for STAMBP than STAMBPL1, despite a 56% sequence homology. This was determined to be a result of key hotspot mutations in the Ub C-terminal tail and the preservation of wildtype Arg-42 [52].

Alternatives to the method used by the Sidhu group have been used to produce potent UbVs. Early efforts to optimize an inhibitor for USP7 relied on in silico methods to guide phage display library design [128]. A UbV for UCHL1 was designed almost entirely using in silico methods. Hewitt et al. predicted the residues of wildtype Ub necessary to minimize the binding of free energies. Residues that could tolerate a mutation without destabilizing the complex with UCHL1 were systematically mutated to identify variants with improved binding while maintaining selectivity over the closest homolog, UCHL3. The result was UbV^T9F/T66K^ with a specificity index of 33 in favor of UCHL1 [123]. In a follow-up study, the same group developed a binder (UbV^Q40V/T9F/V70F^) with a specificity for UCHL3 over UCHL1 [124], but its selectivity relative to a boarder set of DUBs was not tested rigorously.

### 3.1. Diversifying the UbV Portfolio

First, when considering the vastness of the UPS, only a fraction of the possible UbV targets have been successfully hit. Screening the billions of UbVs in existing libraries will undoubtedly lead to discovering new binders. New randomization strategies for the library design can also help identify novel binders with previously undescribed functions. Further variation of the β1–β2 loop might be key [120,129]. An exciting future for UbVs are those targeting pseudo-DUBs to evaluate their roles as scaffolds in macromolecular complexes [31].

Second, a focus should be placed on developing UbVs that bind alternative sites. The intra- and intermolecular modulation of DUB activity is well-characterized [17,24]. USP7 is autoactivated by its C-terminal Ubl domain (HUBL-45) [130]. High-affinity UbVs able to outcompete HUBL-45 for binding to the catalytic domain may facilitate constitutive modulation.

Third, poly-UbVs for DUBs have not yet been appreciably explored. The USP15-targeting di-UbVs 15.1/D and 15.D/1 illustrate how a multisite approach can increase both the affinity and specificity [120]. Strand-swapped dimeric UbVs also point to the usefulness of this approach [113,114,119,120]. Such poly-UbVs may be useful in cases where mono-UbVs lack selectivity for homologous DUBs. Additionally, chain length and linkage specificity are the major determinants of activity amongst certain DUB families [17]. UbVs with a specificity for distinct Ub-binding sites (e.g., S2, S1, S1’, and S2’) may be developed and sequentially incorporated into poly-Ub substrates. This will further our understanding of chain/linkage-specific DUBs. It is noteworthy that all eight linkage types are attainable by enzymatic or (semi)synthetic methods [76,131,132]. Thus, applying these to the UbV design will greatly expand the capabilities of this technology.

### 3.2. UbV Characterization

UbVs should be treated in a similar manner to chemical probes [82]. Holistic validation of their potency, selectivity, and mechanism of action (MoA) should be provided when possible. We propose the community set standards for the validation of newly developed UbVs using in vitro and in vivo methods. Potency should ideally be quantified using orthogonal biophysical methods and complemented with biochemical evidence (e.g., enzyme activity assays). Target selectivity should, at the minimum, be established against closely related structural homologs or by profiling a larger subset of potentially cross-reactive targets. Expression of the UbV in cells can provide evidence of target selectivity by monitoring the colocalization and pathway-specific phenotypic changes. Affinity purification coupled to mass spectrometry (AP-MS) can be used to confirm the tight binding of UbV and the target. Lastly, characterizing the MoA should be attempted. This can be established by biochemical methods, such as mutagenesis and activity assays. Ideally, these data would be supported by structural information in the form of high-resolution cocrystal structures (Figure 2B).

### 3.3. Intracellular Delivery of UbVs

As described above, target validation for UbVs in live cells or cell lysates is relatively straightforward. Cellular delivery is usually mediated by the transient transfection of a plasmid encoding the UbV of interest or by the generation of inducible stable lines (e.g., viral transduction). Alternatively, purified UbVs conjugated to a reactive group can be added to cell lysates to facilitate activity-based protein profiling (ABPP, discussed below). However, these methods do not permit in vivo delivery in a drug-like manner. As UbVs are cell impermeable, delivery methods are required to gain access to their intracellular targets and to assess their therapeutic potential in live cells.

Methods for delivering biopharmaceuticals have been reviewed previously [133,134,135]. As opposed to virus-mediated transduction, delivering purified proteins, such as UbVs, offers better control of the dosage and pharmacokinetics. Cell-penetrating peptides (CPP) [136] and pore-forming toxins [137] have been used to deliver Ub-based probes intracellularly, the former being more amenable to in vivo applications. Virus-like particles (VLPs) are also a means of protein delivery [138] and can be engineered with distinct cellular tropisms [139,140]. Another opportunity for the intracellular delivery of purified UbVs is by modification with oligonucleotides to generate protein core spherical nucleic acids (proSNAs) that are efficiently endocytosed [141] (Figure 2C). The therapeutic potential of UbVs is clear, but the challenges of intracellular delivery, endolysosomal escape, and target engagement remain.

## 4. Activity-Based Probes: Capturing DUBs in Action

Activity-based probes (ABPs) have long been used as tools to study the mechanism of metabolic and signaling enzymes, including DUBs. When applied to whole proteomes, ABPs can resolve changes to the enzyme activity independent of the expression levels. They have been used to discover new DUBs and determine the structures of Ub-bound DUBs for compound/inhibitor screening and for Ub substrate profiling. Many excellent reviews have summarized their design, optimization, and application [76,78,133,142,143,144].

**Figure 3:**
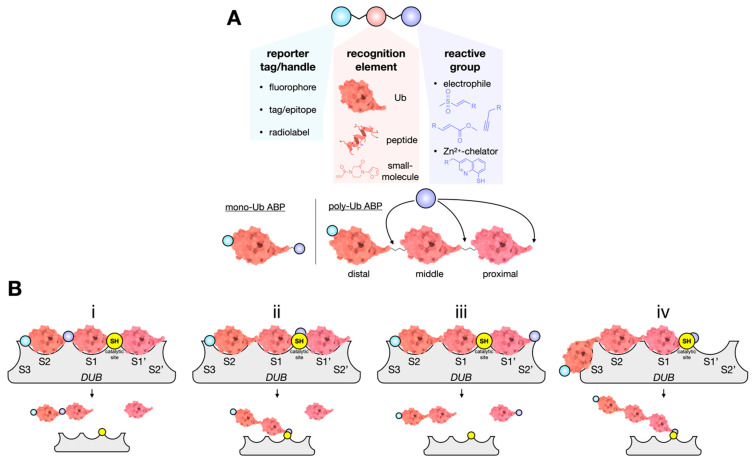
Design of a DUB-targeting ABP. (**A**) Many combinations of the reporter tag, recognition element, and reactive group are possible. For mono-Ub ABPs, the reactive group is placed on the C-terminus. For poly-Ub ABPs, an electrophilic warhead can be strategically placed depending on the DUB cleavage mechanism. Presently, zinc--chelating ABPs for metalloDUBs have only been generated using a C-terminal reactive (chelating) group. (**B**) For poly-Ub ABPs (a tri-Ub ABP, as shown), the substrate must first be recognized and bound by the DUB, a mechanism dependent on the Ub linkage type, chain length, and binding sites (S3−S2’ depicted). Productive catalysis takes place when the scissile bond spans the catalytic site between the S1 and S1’ Ub-binding sites. As shown, only when the reactive group is oriented appropriately will activity-based labelling occur (see ii and iv). Schematic representation of some, but not all, possible outcomes for a tri-Ub ABP bound to a DUB with multiple Ub-binding sites.

These adaptable molecules incorporate three functional elements: (1) a recognition element that endows the target specificity, (2) a reactive moiety (or warhead) that covalently reacts with a target catalytic residue, and (3) a reporter tag (or handle) to facilitate the detection and/or enrichment of the ABP-labeled target enzyme (Figure 3A). The most used recognition element for DUB proteases is Ub. Reporter tags are placed at the N-terminus, thus not interfering with the DUB-Ub interaction. The reactive moiety replaces the C-terminal Gly-76 residue to be positioned within the active site and in proximity to the catalytic residues. Most DUBs are papain-like cysteine proteases, meaning an appropriate electrophile will target conserved nucleophilic cysteines and react to form a reversible or irreversible covalent adduct. However, in lieu of a catalytic triad, metalloDUBs use a catalytic water molecule and do not form a covalent tetrahedral intermediate with the substrate [4]. ABPs that target metalloDUBs require specialized chemistry [145]. Hameed et al. developed a zinc chelating ABP that forms a tight complex with STAMBP and STAMBPL1 and inhibits the activity of the proteasomal DUB Rpn11 (PSMD14 in humans). The chelator, 8-mercapto quinolone (8MQ), when attached to the Arg-74 of Ub, is positioned for zinc binding within the metalloDUB active site [146].

A mono-Ub C-terminal electrophile that binds the S1 site is the simplest DUB ABP. To study linkage specificity, poly-Ub ABPs that occupy multiple Ub-binding sites have been developed [143,147]. The design of such probes requires careful consideration of the Ub linkage type, the linker between Ub molecules, and location of the reactive moiety (Figure 3B). We recently synthesized di- and tri-Ub hybrid ABPs to determine the mechanism of USP9X [39]. We found that *endo*-cleavage was preferred for K11 and K63 but not K48-linked tri-Ub ABPs that contained a non-cleavable distal linkage and a native, cleavable proximal isopeptide linkage. Interestingly, K48-linked tri-Ub is exclusively processed by the *exo*-cleavage mode. The ABP that resolved this was a tri-Ub containing a distal Michael acceptor warhead and a native, cleavable-proximal isopeptide bond. These data suggest that USP9X poly-Ub processivity is linkage-dependent and more rapidly deconjugates both K11 and K63 rather than K48-linked chains [39].

### ABP Specificity

Using Ub as the recognition element affords DUB-targeting ABP broad selectivity. To afford a greater target specificity, UbV ABPs have been utilized in the past [122,123,124]. Inspection of the available DUB-targeting UbVs suggests many would work well as ABPs, while others may not. Despite binding the S1 Ub-binding site, when UbV8.2 is bound to USP8, it is rotated relative to the conformation expected for wildtype Ub [103]. Consequently, the C-terminal tail does not extend into the catalytic pocket. In this case, it is reasonable to suspect a C-terminal electrophile would not gain access to the catalytic cysteine.

Instead of a full-length Ub, smaller recognition elements such as Ub-derived peptides and small molecule inhibitors have also been used [89,148,149,150]. Not only does this assist with cell permeability, but it can be a powerful method for screening off-targets in the drug discovery pipeline [90,133,148,149]. Such as that described for UbV8.2, one must consider the orientation of the recognition element when bound to its target, as this will directly influence the proximity of the reactive group to the catalytic site. Another consideration is that the catalytic residues of some apo-DUBs are misaligned, and the rearrangement is driven allosterically upon substrate binding. When misaligned, the catalytic cysteine is less polarized and, therefore, less reactive. This can have a negative effect on the ABP performance. Small molecules may not occupy the vast surface area required to induce the conformational changes for alignment of the catalytic residues. In essence, the chosen peptide or small molecule must be able to target the apoenzyme catalytic site [77].

The choice of reactive group (cysteine-reactive electrophile) must be carefully considered, as different chemistries will influence the reactivity and selectivity. This was demonstrated recently when comparing the reactivity of a UCHL1-targeting ABP bearing either a propargyl amide (PA) or vinyl methyl ester (VME) warhead [123]. Furthermore, reactions with cysteine residues are fraught with potential nonspecific products based on the prevalence of biological thiols [151]. DUB-targeting ABPs have not only been shown to label non-DUB proteins but also non-catalytic DUB cysteines [39,143,152,153]. A K11-linked di-Ub ABP with an internal VME warhead conjugated USP9X at Cys-1808, a noncatalytic blocking loop (BL) residue. This could not be repeated with K48- or K63-diUb ABPs or mono-Ub ABPs [39]. The significance of this is yet to be fully resolved. It is enticing to posit that the underlying mechanism is conserved amongst other BL-containing DUBs, but sequence analyses show that Cys-containing BLs are uncommon.

## 5. Unifying Technologies to Study DUBs at the Cellular Level

Cell-based assays that successfully address a particular biological question depend on a reliable method with an unadorned readout. For DUBs, a major goal is to establish a more complete understanding of the up- and downstream effectors, interacting partners, substrates, and context-dependent specific activity. To mitigate artifacts is critical and requires highly selective tools and unintrusive methods. In this final section, we discuss genetic and pharmacological approaches to studying DUBs at the cellular level and how high-quality modulators are incorporated to bolster the results.

### 5.1. DUB-Omics

Omics-level analyses of PPIs give a broad view of the molecular networks. Laundry lists of high-confidence interactors serve as great starting points for characterizing novel interactions and their associated biological significance. Classical proteomics workflows rely on AP-MS to identify co-purifiers of an endogenous or overexpressed protein of interest. Using AP-MS, the BioPlex project expanded the known human interactome [154,155]. The resulting datasets provided interaction networks for many DUBs [156]. Sowa et al. also performed AP-MS interactomics, with a focus instead on 75 DUBs. At that time, this was the most comprehensive analysis of the DUB interaction landscape, revealing their cellular distribution and functional overlap [26]. Similarly, the intracellular distribution of DUBs in fission yeast was determined using endogenous GFP fusions monitored by confocal microscopy and validated by AP-MS-based proteomics [157]. In another study, an analysis of the host–virus interactome upon SARS-CoV-2 infection was examined systematically by expressing viral proteins in human lung carcinoma cells. Notably, SARS-CoV-2 ORF3 and ORF7a proteins interacted with USP8 and USP34, respectively [158]. ORF3 is ubiquitinated by host E3 ligases and localizes to the endolysosomal compartment, where it impinges on autophagy [159]. This places it directly in the locale of USP8 and suggests the hijacking of host DUB machinery for viral function.

Inherent to the AP-MS methods are biases against transient interactions, the isolation of only soluble proteins, and the non-physiological association of once-segregated (compartmentalized) proteins upon cell lysis and dilution. To circumvent this, proximity-dependent biotinylation coupled to MS (PDB-MS or BioID) was developed [160]. While the overarching theme of resolving PPIs in situ remains constant, the method of BioID has improved over time [161,162,163,164]. Using this technique, Gingras Lab mapped the compartmentalized landscape of the entire human cell (HEK293), which included the distribution of many DUBs therein [165]. However, on a few occasions, DUBs have been chosen as BioID baits—that is, fused to a promiscuous biotin ligase (BirA^*^). A systematic study of DUB interactomes by BioID would be an indispensable asset to the community. Using BioID, USP35 isoforms 1 and 2 were shown to be anti- and proapoptotic, respectively. As baits, isoform 1 was found to associate with cytosolic proteins, whereas isoform 2, containing a transmembrane domain, was associated with ER membrane proteins [27]. The distribution of the USP35 isoforms is strikingly similar to that observed for the isoforms of USP19 [166].

DUB interactors can be divided into three classes. (1) Constitutive binding partners, often as part of larger macromolecular complexes, e.g., USP22/SAGA-DUBm [167] and CSN5/COP9 signalosome [168]; (2) low-affinity, transient binding partners, e.g., the USP19/SIAH complex [169] and USP8/NRDP1 [170], or protein modifiers such as kinases, e.g., USP10/AMPKα [171]; and (3) ubiquitinated substrates that bind DUBs by virtue of the Ub modification. It is a challenge to distinguish between these on an omics level. Class 1 interactors can be isolated by AP-MS, but classes 2 and 3 are more difficult. In situ proximity labeling is particularly useful in these cases. A recent example of uncovering class 3 interactors was demonstrated by the Clague and Urbé groups for the mitochondrial integral membrane DUB USP30. Using compound FT385, a probe-quality covalent USP30 inhibitor, they conducted comparative analyses of quantitative proteomics data and showed that USP30 inhibition had little effect on the global proteomics profile but a marked increase of ubiquitinated voltage-dependent anion channel family proteins (porins of the mitochondrial outer membrane) [88]. More recently, Bushman et al. used chemical probe-quality small molecule inhibitors of USP7 to reveal novel substrates. The proteomics approach involved treating myeloma cells with the noncovalent inhibitor XL188 [98] or the covalent inhibitor XL177A [97] (and enantiomeric control compounds) and quantifying protein abundances by a tandem mass tag MS [172]. This study demonstrates that merging DUB-omics with pharmacological interventions is a powerful technique for validating DUB inhibitors, as well as for probing the DUB interactome.

### 5.2. Genetic and Pharmacological Modulation Reveals DUB Biology

The genetic interference of target genes using RNAi or clustered regularly interspaced short palindromic repeat (CRISPR) technology has become routine in regular laboratories. The challenge of genetic interference is that the encoded proteins are knocked down or removed from cells in their entirety. Since most DUBs have domains other than the catalytic domain that could play essential roles in target localization, trafficking, substrate recruitment, PPIs, or scaffolding, knockdown or knockout of the protein can often have unexpected outcomes that differ from the inhibition of the enzymatic activity. The drawback can be partially salvaged by using CRISPR base editing or mutagenesis techniques [173,174]. This was nicely demonstrated in a study of the UCH family member BAP1. In liver organoids, missense, nonsense, indels, and frameshift mutations were introduced using CRISPR editing to explore its tumor-suppressor function [175]. In another case, the Summers group used CRISPR to study the histone DUB USP16 [176]. The homozygous deletion of *USP16* is embryonic-lethal in mice [177]; therefore, two rounds of CRISPR-mediated mutagenesis were used to introduce homozygous inactivation mutations. In doing so, the authors found that the human monocytic leukemia THP-1 cell line can develop alternative pathways to escape the inactivation mutations of USP16, suggesting that the function of USP16 is potentially redundant, and another unstudied DUB may compensate for the loss of function. The result argues against the therapeutic value of targeting USP16. However, the inactivating mutation introduced a nonsense stop codon that truncated the C-terminal catalytic domain of USP16 while leaving the N-terminal zinc finger domain. It is not clear whether pharmacological interference will lead to the same outcome as that of the genetic interference described here. No small molecule inhibitors have been reported for USP16. However, there is one UbV [122] with apparently good selectivity that could render a viable comparison.

There are still very limited examples of introducing inactivated mutations in DUBs using CRISPR editing technology. It is prudent to compare the results from genetic interference with those from pharmacological interference, which targets the enzymatic activity of DUBs specifically. Done in parallel, these efforts would validate the functional roles of the DUBs under study. In a recent example, the Dirks and Angers groups used genome-wide CRISPR screening and identified USP8 as one of the vulnerable genes from patient-derived glioblastoma stem cells and then validated USP8 to be an actionable therapeutic target using the lentiviral delivery of a UbV inhibitor [178].

## 6. Conclusions

DUB research remains a field ripe with opportunity. Both small molecule inhibitors and biological probes, such as ABPs and UbVs, have proven to be powerful tools in studying the biology of the DUB family of enzymes. While small molecules are the gold standard therapeutic scaffold, UbVs are critically important to leverage to expand the available DUB modulators. The demonstration of the druggability of DUBs, especially that of USP7, which has a canonical USP fold lacking the insertions commonly seen in many USP family members, and the recent publications of several high-quality inhibitors for other USPs, shall attract renewed interest in drug discovery for this challenging but important family of proteins. So far, there have been very few chemical probe-quality inhibitors. We observed approximately 15, hitting just 6 of the >100 DUB targets. This constitutes only three of the seven families. Many new structures of DUBs have been reported in recent years, and artificial intelligence-driven, structure-guided small molecule drug discovery would be a particularly interesting application.

Given the naturally large Ub-binding pocket of DUBs, the UbV technology is especially suited for finding potent DUB inhibitors. This technology is suitable for generating inhibitors rapidly and cost effectively. These DNA-encoded inhibitors can be delivered using a lentiviral system or plasmid-mediated transfection to study the cellular function of the cognate DUBs. However, the direct delivery of cell impermeable, purified UbVs needs to be addressed further. The recent successful nanoparticle-encapsulated mRNA vaccine for COVID-19 is another potential UbV delivery technique that has not been tried and would be worth exploring.

Ubiquitination is an extremely complicated PTM compared to others, such as phosphorylation, methylation, and acetylation. A near-complete set of high-quality tool reagents targeting each individual DUB, combined with genetic intervention and proteomics, would be a dream team for studying the fascinating biology of DUBs at the cellular level.

## Figures and Tables

**Table 1:** Potent small molecule DUB inhibitors.

		Characterization					
Compound	Target	IC_50_/*K*_i_/*K*_d_ (nM)	MoA	Structure (pdb)	Negative Control	Validation	Ref.
IMP-1710	UCHL1	38	covalent, slowly reversible		IMP-1711	cell-based assays; dose-response; in vivo target engagement; chemical proteomics	[90]
CSN5i-3	CSN5	5.8	non-covalent	5jog	*R*,*R*-CSN5i-2e	enzyme assays; cell-based assays; dose-response; XRD	[92]
Azaindole derivatives cmpds 4, 6 ^1^	CSN5	90, 60	non-covalent	5m5q ^2^		enzyme assays; NMR; SPR; CellTiter-glo; XRD	[93]
FT671	USP7	52	non-covalent	5nge		mutagenesis; cell-based assays; SPR; MS	[94]
GNE6640	USP7	750	non-covalent	5uqv	GNE-6641	enzyme assays; cell-based assays; NMR; MS; XRD	[40]
ALM-4, ALM-5	USP7	1.5, 22	non-covalent	5n9t ^3^	*ent*-ALM-4	cell-based assays; SPR; target engagement assay; XRD	[95]
Pyrimidinone derivatives ^4^	USP7	6−87 ^4^	non-covalent	6f5h ^5^		enzyme assays; cell-based assays; in vitro ADME assay; XRD	[96]
XL177A	USP7	0.34	covalent		XL177B	enzyme assays; cell-based assays; ABPP-MS; HDX	[97]
XL188	USP7	90	non-covalent	5vs6	XL203C	enzyme assays; cell-based assays; ITC; XRD	[98]
FT709	USP9X	82	non-covalent			cell-based assays; proteomics; SPR; ELISA	[72]
ML323	USP1/UAF1	76	non-covalent		NCGC-959	enzyme assays; cell-based assays; orthogonal gel-based assays	[81]
MF-094	USP30	120	non-covalent		MF-095	enzyme assays; mitophagy assay	[99]
FT385	USP30	1	covalent			enzyme assays; cell-based assays; BLI; ABPP	[88]

^1^ These compounds have a scaffold different from that of CSN5i-3. ^2^ Compound 4 in a complex with CSN5. ^3^ ALM-5 in a complex with USP7. ^4^ Multiple inhibitors of sub-micromolar affinity with the same chemical scaffold were reported. ^5^ Compound 46 in a complex with USP7. ABPP, activity-based protein profiling; ADME, absorption, distribution, metabolism, and excretion; BLI, biolayer interferometry; ELISA, enzyme-linked immunosorbent assay; HDX, hydrogen-deuterium exchange; ITC, isothermal titration calorimetry; MoA, mechanism of action; MS, mass spectrometry; NMR, nuclear magnetic resonance; SPR, surface plasmon resonance; XRD, X-ray diffraction.

**Table 2:** UbVs and their cognate human DUB targets. Studies identify multiple UbVs during the initial screening and optimization. For simplicity, this table summarizes only a subset of the matured DUB binders.

		Characterization						
UbV	Target	IC_50_ (nM)	EC_50_ (nM)	*K*_d_ (nM)	Validation	Specificity ^1^	Co-Structure (pdb)	Ref.
2.3	USP2a	25			phage ELISA; enzyme assays; XRD	++	3v6e	[103]
7.2	USP7	0.91	10.9		phage ELISA; enzyme assays; cell-based assays; AP-MS; GF; XRD	+++		[74]
8.2	USP8	4.8			phage ELISA; enzyme assays; cell-based assays; AP-MS; XRD	+++	3n3k	[103]
10.1	USP10	46.2	39.9		phage ELISA; enzyme assays; cell-based assays	++		[74]
15.1/D	USP15	6.6			ELISA; enzyme assays; cell-based assays; AP-MS	+++	6dj9 ^2^	[120] ^3^
M20	USP16				in silico, enzyme assays; cell-based assays; AP-MS; ABP profiling	+++		[122]
21.4	USP21	2.4			phage ELISA; enzyme assays; cell-based assays; AP-MS; XRD	+++	3mtn	[103]
25.1	USP25	130			competitive phage ELISA	++		[117]
28	USP28	15			competitive phage ELISA; cell-based assays	++		[117]
37.3	USP37	190			competitive phage ELISA	++		[117]
T9F/T66K	UCHL1	2200		1300	in silico; BLI; enzyme assays; cell-based assays	+		[123]
Q40V/T66K/V70F	UCHL3	4		49	in silico; BLI; enzyme assays; cell-based assays	+		[124]
SP.3	STAMBP	9.8		60	phage ELISA; enzyme assays; ITC; XRD	+	7l97 ^4^	[52]
BR.1	BRISC				phage ELISA	++		[103]
B1.1	OTUB1		11,100	20	phage ELISA; enzyme assays; XRD	++	4i6l	[103]
OTUD1	OTUD1	10			competitive phage ELISA	++		[117]
Ataxin3.2 & 3.3	ATXN3	20			competitive phage ELISA	++		[117]

^1^ Specificity determined relative to one structural homolog in vitro is denoted by +; specificity established across a panel of purified targets in vitro is denoted by ++; specificity further established by proteomic profiling of UbV co-purifiers is denoted by +++. ^2^ UbV.15.D in a complex with the USP15 DUSP domain. ^3^ This study identifies multiple potent UbVs targeting different domains of USP15. ^4^ UbV^SP.1^ in a complex with the STAMBP homolog STAMBPL1. AP-MS, affinity purification coupled to mass spectrometry; GF, gel filtration.

## Data Availability

Not applicable.

## Abbreviations

ABP, activity-based probe; AMC, 7-amido-4-methylcoumarin; BLI, biolayer interferometry; DUB, deubiquitinase; CPP, cell-penetrating peptide; CRISPR, clustered regularly interspaced short palindromic repeat; ELISA, enzyme-linked immunosorbent assay; GF, gel filtration; ITC, isothermal titration calorimetry; JAMM, JAB1/MPN/MOV34 domain-associated; MINDY, motif interacting with the Ub-containing novel DUB family; MJD, Machado-Joseph domain-containing proteases; MoA, mechanism of action; MS, mass spectrometry; OTU, ovarian tumor; PA, propargyl amide; PDB-MS, proximity-dependent biotinylation coupled to MS; PPI, protein–protein interaction; MS, mass spectrometry; PTM, post-translational modification; SPR, surface plasmon resonance; SNA, spherical nucleic acids; Ub, ubiquitin; UBD, Ub-binding domain; Ubl, Ub-like; UbV, Ub variant; UCH, Ub C-terminal hydrolase; UIM, Ub-interacting motif; UPS, Ub proteasome system; USP, Ub-specific protease; VLP, virus-like particles; VME, vinyl methyl ester; XRD, X-ray diffraction; ZUFSP, zinc finger with the UFM1-specific peptidase domain protein.
